# Surgical Oncology: Multidisciplinarity to Improve Cancer Treatment and Outcomes

**DOI:** 10.3390/curroncol28060379

**Published:** 2021-11-04

**Authors:** Jörg Kleeff, Ulrich Ronellenfitsch

**Affiliations:** Department of Visceral, Vascular and Endocrine Surgery, University Hospital Halle (Saale), Martin-Luther-University Halle-Wittenberg, 06120 Halle (Saale), Germany; ulrich.ronellenfitsch@uk-halle.de

Surgical oncology is commonly regarded as the field of surgery dealing with cancer. In the 19th century, with the advent of modern surgical and anesthesiological principles, surgeons slowly dared into the removal of tumors and the affected organs. The notion arose that malignancies can be cured by resecting them with a sufficiently wide tissue margin, which culminated in the approach pioneered by William Steward Halsted, who performed extensive resections for breast cancer not only comprising a mastectomy, but also the pectoralis muscles and the entire axillary lymphatic tissue, thereby reducing recurrence rates substantially [1]. Only decades later it was shown that the radicality of this approach is actually not superior to a more contained and targeted operation in terms of recurrence and survival [2]. Over the years, gastrectomy, colectomy, esophagectomy, and pancreatectomy were all pioneered and subsequently adopted into clinical practice, thus offering a chance of cure for patients with gastrointestinal malignancies. In the 1960s, Turnbull propagated the “no-touch isolation technique” for colorectal cancer resections, in which a high lymphovascular tie early during the operation and a lymphatic clearance are performed while avoiding any manipulation of the tumor. This approach proved superior to hitherto used resection techniques regarding survival in retrospective analyses [3]. The concept of systematic lymphadenectomy of the supposed lymphatic drainage area of a given tumor was thus established and subsequently further developed into techniques such as total mesorectal excision [4], complete mesocolic excision [5], or the TRIANGLE operation for pancreatic cancer [6]. Another approach, which has rendered the surgical treatment of diseases such as melanoma and breast cancer much more individualized, is sentinel lymph node biopsy and selective application of radical lymphadenectomy [7]. The last two decades were marked by the adoption of minimally invasive approaches in oncological surgery. For colorectal and esophageal cancer, the equivalence of laparoscopic or thoracoscopic to open resections regarding recurrence and survival and their superiority regarding early postoperative outcomes have been demonstrated [8,9,10]. At present, several trials assess the equivalence or superiority of robotic surgery for esophagectomy or rectal resection [11,12].

However, surgery is only one of the three pillars on which the treatment of solid malignancies rests, the other two being systemic therapy and radiotherapy (as well as other local ablative therapies). For some malignancies, such as head and neck cancer or anal carcinoma, radiotherapy alone or in combination with chemotherapy can be curative, thus obviating the need for mutilating surgery [13,14]. For many other solid tumors, the administration of chemotherapy, radiotherapy, or both, as a preoperative, postoperative, or perioperative regimen, is associated with relevant survival benefits. Outstanding examples are gastroesophageal adenocarcinoma, for which perioperative chemo- or chemoradiotherapy have improved five-year survival by about ten percent [15], and colon cancer, for which postoperative chemotherapy has led to substantial survival improvements [16]. Traditionally systemic treatment consisted of cytotoxic drugs, however, by now targeted agents have been successfully employed in perioperative treatment as well, such as imatinib before and after resection of gastrointestinal stroma tumor [17,18]. Lately, immunotherapeutic agents effecting, for example, the PD-1/PD-L1 blockade, have also been used in the perioperative setting, with melanoma being an emblematic example for the efficacy of this approach [19]. In addition, systemic therapy and external beam radiotherapy, intraperitoneal, or liver-directed chemotherapy and intraoperative radiotherapy are also established treatments for selected tumor entities [20,21,22].

The knowledge of all the described treatment options is indispensable for any surgical oncologist. To be able to offer cancer patients the best available treatment in terms of not only technical features but also the indication for and timing of an operation, surgical oncologists need to possess a broad and constantly updated knowledge of systemic and radiotherapeutic treatments and their possible combinations with surgery. Such knowledge can be acquired through formal training programs, as well as through a variety of courses and, lastly, also through self-study. Whereas in North America, surgical oncology has become a board certified specialty [23], in other health care systems this is rarely the case. However, professional societies or academic or private entities offer training programs and award titles or degrees of varying content and denomination. Standardization of curricula and degrees would be desirable in order to harmonize knowledge and to allow recognition across borders.

The ideal treatment recommendation for a given patient with cancer cannot be reached by a surgeon or surgical oncologist alone, but should be agreed on in a multidisciplinary manner. An ideal format for this purpose are tumor conferences. In such meetings, representatives of all disciplines involved in cancer treatment gather on a regular basis and reach a consensus on what would constitute the best treatment taking into account the latest evidence, as well as preferences and characteristics of the patient. This approach not only provides a more solid basis for discussing possible treatments with patients, but studies also suggest that it might improve treatment outcomes, albeit on a low evidence level [24,25].

Surgical oncology is a field that has experienced profound developments over the last decades. For any surgical oncologist, a broad knowledge and extensive skills of all aspects of cancer care and the motivation to collaborate with all members forming part of multidisciplinary teams are key for providing patients with the best possible treatment for their disease.

## Data Availability

Not applicable, study did not report any data.
